# Programmed vs. Thirst-Driven Drinking during Prolonged Cycling in a Warm Environment

**DOI:** 10.3390/nu14010141

**Published:** 2021-12-29

**Authors:** David Jeker, Pascale Claveau, Mohamed El Fethi Abed, Thomas A. Deshayes, Claude Lajoie, Philippe Gendron, Martin D. Hoffman, Eric D. B. Goulet

**Affiliations:** 1 Faculty of Physical Activity Sciences, University of Sherbrooke, Sherbrooke, QC J1K 2R1, Canada; david.jeker@usherbrooke.ca (D.J.); pascale.claveau2@usherbrooke.ca (P.C.); Mohamed.El.Fethi.Abed@USherbrooke.ca (M.E.F.A.); thomas.deshayes@usherbrooke.ca (T.A.D.); 2 Research Center on Aging, University of Sherbrooke, Sherbrooke, QC J1H 4C4, Canada; 3 Department of Physical Activity Sciences, Université du Québec à Trois-Rivières, Trois-Rivières, QC G8Z 4M3, Canada; claude.lajoie@uqtr.ca (C.L.); philippe.gendron@uqtr.ca (P.G.); 4 Department of Physical Medicine & Rehabilitation, University of California Davis, Sacramento, CA 95817, USA; mdhoffman@ucdavis.edu; 5 Ultra-Endurance Sports Science & Medicine, Duluth, MN 55811, USA

**Keywords:** cycling, hydration, endurance performance, thirst, fluid balance, prolonged exercise

## Abstract

We compared the effect of programmed (PFI) and thirst-driven (TDFI) fluid intake on prolonged cycling performance and exercise associated muscle cramps (EAMC). Eight male endurance athletes (26 ± 6 years) completed two trials consisting of 5 h of cycling at 61% $\dot{V}O_{2\mathrm{peak}}$ followed by a 20 km time-trial (TT) in a randomized crossover sequence at 30 °C, 35% relative humidity. EAMC was assessed after the TT with maximal voluntary isometric contractions of the shortened right plantar flexors. Water intake was either programmed to limit body mass loss to 1% (PFI) or consumed based on perceived thirst (TDFI). Body mass loss reached 1.5 ± 1.0% for PFI and 2.5 ± 0.9% for TDFI (*p* = 0.10). Power output during the 20 km TT was higher (*p* < 0.05) for PFI (278 ± 41 W) than TDFI (263 ± 39 W), but the total performance time, including the breaks to urinate, was similar (*p* = 0.48) between conditions. The prevalence of EAMC of the plantar flexors was similar between the drinking conditions. Cyclists competing in the heat for over 5 h may benefit from PFI aiming to limit body mass loss to <2% when a high intensity effort is required in the later phase of the race and when time lost for urination is not a consideration.

## 1. Introduction

Exercise-induced dehydration may sometimes have a negative impact on endurance performance (EP) [1]. Accordingly, it is recommended for athletes to consume sufficient fluid during prolonged exercise. Several studies have shown that thirst-driven fluid intake (TDFI) may be all that is necessary to optimize EP [2,3,4]. Distinctively, the American College of Sport Medicine (ACSM) recommends to program fluid intake (PFI) to prevent a body mass loss ≥2% during exercise [5]. Nevertheless, a slight mismatch between the sweat rate and fluid intake during prolonged exercise could result in excessive over- or under-drinking, which may potentially lead to health problems or impaired EP. Undeniably, exercise duration is a key factor to consider with regards to fluid intake.

Results of a recent meta-analysis have shed light on the impact of these two hydration strategies on EP during exercise of 60 to 127 min in duration. Indeed, Goulet and Hoffman [6] demonstrated that PFI and TDFI have a similar effect on EP. To our knowledge, however, no study has simultaneously examined the effect of PFI and TDFI on EP during exercise lasting more than 127 min. Hence, hydration recommendations regarding those two strategies are, at best, speculative when it comes to prolonged (>4–5 h) exercise duration.

During prolonged exercise, each of those hydration strategies might be associated with potential advantages and disadvantages. Fluid replacement is highly likely to be greater with PFI than TDFI [7]. Therefore, PFI might decrease cardiovascular and thermal strain and perceived exertion more than TDFI. However, a greater volume of fluid ingested with PFI might lead to abdominal discomfort. PFI is also likely to be associated with more mictions [8] than TDFI during prolonged exercise; however, the psychological advantage, i.e., placebo effect, provided by the PFI might be particularly important during prolonged exercise in the heat, which may be to the disadvantage of TDFI.

The development of exercise-associated muscle cramps (EAMC) is a practical concern with potentially negative consequences for athletes during prolonged exercise. EAMC may be caused either by dehydration or sodium depletion/dilution [9], or by fatigue-induced abnormal spinal reflex activity [10]. On the one hand, PFI might lead to a greater dilution of electrolytes than TDFI during prolonged exercise whereas, on the other hand, more dehydration with TDFI might lead to a greater muscular fatigue than PFI through, for instance, greater thermal stress. No studies to date have examined the impact of PFI and TDFI on the prevalence of EAMC and the uncertainty of their effect encourages research.

From a health perspective, there exists a legitimate preoccupation as to whether PFI might put athletes at a greater risk for the development of hyponatremia (serum sodium < 135 mmol∙L^−1^) than the use of TDFI during prolonged exercise [11]. In fact, whereas the rate of fluid intake with TDFI is regulated such to maintain plasma osmolality and sodium levels within an optimal window, that associated with PFI rests on a variable that is not acutely physiologically regulated, i.e., body mass [12]. However, although Barr, Costill and Fink [8] showed that water consumption aimed at replacing sweat losses during 6 h of cycling in the heat did decrease blood sodium concentration, the magnitude of change was not sufficient to induce hyponatremia. 

The goal of the current study was to examine the effect of PFI and TDFI during a 5 h, moderate intensity fixed-workload cycling bout followed by a 20 km time-trial (TT) on EP, selected physiological functions and perceptual sensations, blood sodium concentration and the prevalence of EAMC. It was hypothesized that fluid balance would be better maintained with PFI than TDFI, which would reduce cardiovascular and thermoregulatory stress and blood sodium concentration. Perceived exertion would not be different between the conditions, TT performance would be faster with TDFI than PFI, there would be more stops for mictions with PFI than TDFI and none of the conditions would induce hyponatremia. Both hydration strategies were expected to have a similar effect on EAMC.

## 2. Materials and Methods

### 2.1. Participants

Eight well-trained male endurance athletes completed the study. Their mean (±SD) age, height, body mass, peak oxygen consumption ($\dot{V}O_{2\mathrm{peak}}$) and body fat percentage were 26 ± 6 years, 178 ± 7 cm, 71 ± 6 kg, 4.7 ± 0.3 L min^−1^ or 67 mL ± 4 mL·kg^−1^·min^−1^, and 8 ± 2%, respectively. Participants provided verbal and written informed consent to participate in this study, which was approved by the CIUSSS Estrie-CHUS Ethical Committee (# 2019-2837). A total of 11 athletes were initially recruited, but two were excluded because they did not meet the minimal criterion of 55 mL∙kg^−1^∙min^−1^ and another withdrew during his first experimental trial. 

### 2.2. Pre-Experimental Visit

Participants first underwent a preliminary visit to (1) assess physical characteristics and body anthropometry, (2) measure $\dot{V}O_{2\mathrm{peak}}$, using an automated gas analyzer and an incremental cycling test to exhaustion (starting at 100 W with 25 W increments every 2 min until exhaustion) and (3) thoroughly familiarize themselves with the protocol used to induce muscle cramps. 

### 2.3. Pre-Experimental Protocol

Participants were instructed to write down their food and fluid intake (time of the day + quantity) over the last 48 h prior to the upcoming familiarization trial, and to reproduce this diet prior to the experimental trials. Moreover, participants were required to go to bed the night prior the familiarization trial and experiments at the same time and, for the last 24, 48 and 72 h prior to these visits, repeat the same training or rest, avoid sports supplements and stop lower limbs strength training, respectively. One hour prior to going to bed the night before the familiarization trial and experiments, as well as 1 h prior to these visits, participants were required to drink 250 mL of water and then, remain fasted. 

### 2.4. Familiarization Trial

Within 1 to 10 days following the pre-experimental visit, participants completed a familiarization trial consisting of 2 h of moderate, fixed-intensity (60% of $\dot{V}O_{2\mathrm{peak}}$) cycling exercise followed by a 20 km TT. During this trial, participants consumed water according to thirst sensation. The exercise period was performed in an environmental chamber set at 30 °C and 35% relative humidity, with 500–600 W m^−2^ of radiance and 3 large fans in front of the bicycle blowing air at the whole-body level at 25–30 km∙h^−1^. The goal of this trial was (1) to familiarize participants with the environmental conditions and experimental procedures to be used during the experiments, (2) to confirm the workload for the fixed-workload cycling periods, corresponding to a $\dot{V}O_{2}$ of about 60% $\dot{V}O_{2\mathrm{peak}}$, (3) to minimize any learning effect, and (4) to estimate the water to be consumed during the PFI trial to maintain body mass loss at 1%. This percentage of body mass loss was chosen to provide an assurance that the recommended threshold of 2% would not be crossed. The amount of water to be consumed during the PFI trial was specifically estimated for the fixed-workload exercise and 20 km TT using, for the former, the hourly rate of body mass loss during the fixed-workload exercise and, for the latter, the body mass lost during the 20 km TT. 

### 2.5. Experimental Trials

**Figure 1:**
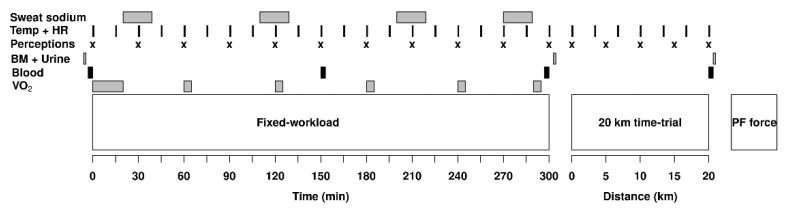
Timeline for the experimental procedures showing when the measurements of skin and rectal temperature (Temp), heart rate (HR), perceptions of thirst, effort, heat stress and abdominal discomfort (Perceptions), body mass (BM), urine specific gravity and osmolality (Urine), hematocrit, hemoglobin, sodium (Blood), sweat sodium concentration (Sweat sodium) and oxygen consumption ($\dot{V}O_{2}$) were taken. The experiment was divided in three phases, a 5 h fixed-workload exercise period, a 20 km time-trial and a plantar flexors (PF) force measurement protocol.

For 11 to 21 days following the familiarization trial, participants underwent, using a randomized crossover protocol, a first experimental trial followed by a second one, 14 to 28 days later. They consisted of 5 h of fixed-workload cycling at a power output corresponding to 61 ± 4% $\dot{V}O_{2\mathrm{peak}}$ followed, 5 min later, by a 20 km TT and then, another 5 min later, by the cramp induction protocol, under the same environmental conditions as those used during the familiarization trial. This exercise protocol was chosen to mimic the demands of a typical road-cycling race of 5 to 6 h in duration [13]. The delays were standardized to 5 min to allow for urine collection and post-void body mass measurement. The timeline for the experimental trial is presented in Figure 1. EP was evaluated from two angles: (1) based solely on TT performance and (2) from a practical perspective, where the total time taken to urinate during exercise was also considered. During the TT, participants had access to their power, distance, elapsed time and speed from a computer screen set in front of them.

### 2.6. Fluid Intake and Urine Collection

During exercise, participants consumed either water according to thirst sensation (TDFI) or, during PFI, in fixed volumes every 15 min. Four opaque bottles (Camelbak podium chill, Camelbak, Petaluma, CA, USA) containing 4 °C water were brought into the environmental chamber at the start of every hour. For the PFI trial, the amount of water to be drunk every hour was equally divided into the bottles and the participants had 15 min to empty each bottle. For TDFI, the water volume consumed was measured every 15 min and bottles were swapped for a full one before they were empty to ensure a constant access to water and to deter the participants from estimating their intakes. In the event that a participant was incapable of drinking the quantity of fluid required to maintain body mass loss at ~1%, the volume to be consumed was subsequently adjusted to reduce fluid replacement by an additional 0.5% of the body mass, until an equilibrium was achieved, if required. Water temperature inside the bottles was measured at the end of each hour for the TDFI condition only as there was normally no water left for the PFI condition. 

Urine was collected during exercise and post-void body mass was measured prior to the experiments, prior to the TT and immediately following the cramp induction protocol. For the PFI trial, urine produced during exercise in excess of the amount measured during the familiarization trial was compensated for by an extra amount of water in order to maintain body mass loss as close as possible to 1%. This amount of water was added to the bottles and consumed during the following hour of exercise. If an athlete needed to stop to urinate, the 5 h ride was paused and resumed after the break so that the same amount of work was completed in both experiments. The time for pausing was recorded and the participants were instructed to proceed as fast as possible while making sure all the urine was discharged in the portable urinal. 

### 2.7. Physiological Functions, Blood and Urine

Measurements were made of heart rate and skin and rectal temperatures every 15 min throughout the fixed-workload exercise period and every 5 min during the TT, $\dot{V}O_{2}$ during 20 min upon the start of exercise and then at min 60, 120, 180, 240 and 290 of the fixed-workload exercise period for 5 min, and subjective perceptions at the start of exercise, every 30 min thereafter and every 5 km during the TT. Capillary blood was collected on 4 occasions during the experiments, i.e., immediately before (after a 10 min rest period sitting on the bicycle), in the middle and at the end of the 5 h fixed-workload exercise period and immediately following the TT. Urine specific gravity was measured with a digital refractometer (PAL-10S; Atago, Bellevue, WA, USA) and osmolality using the freezing point depression technique (Micro Osmometer, Osmette, Precision Systems Inc., Natick, MA, USA).

### 2.8. Nutrition and Entertainment

Participants were provided with 1 g carbohydrate (CHO)∙kg body mass^−1^∙h^−1^ in the form of caffeine-free energy gels (GU Roctane, GU Energy Labs, Berkeley, CA, USA) throughout the exercise period [14]. The gels contained either 125 mg or 180 mg of sodium, and the same flavors were provided in the same order during the experimental trials and were distributed at a personalized fixed time interval based on the athlete’s body mass. Participants could listen to music during the 5 h fixed-workload exercise and TT; however, during the TT, the same playlist was required to be used for both experimental trials [15]. Participants were also allowed to watch videos on their own electronic devices only during the 5 h fixed-workload exercise period.

### 2.9. Physical Characteristics and Body Anthropometry

Measurements were made of post-void body mass using a BX-300 + scale (Atron Systems, West Caldwell, NJ, USA), height with a wall stadiometer and body composition using DXA (Lunar Prodigy DXA, GE Healthcare, Madison, WI, USA). 

### 2.10. Environmental Conditions

Temperature, relative humidity and dew point inside the environmental chamber were recorded at 1 min intervals throughout the experiments with a data logger (DVTH, Supco, Allenwood, NJ, USA). Wind speed was measured with a digital anemometer (DAF800, General Tools & Instruments, Secaucus, NJ, USA) and radiance with a pyranometer (Apogee SP-110-SS, Apogee, Logan, UT, USA).

### 2.11. Oxygen Consumption and Respiratory Exchange Ratio

Oxygen consumption and respiratory exchange ratio were measured using an automated expired gas analysis system (Cosmed Quark CPET, Cosmed, Chicago, IL, USA) which, prior to all use, had been calibrated with gases of known concentration. 

### 2.12. Cycling Exercise

The same electromagnetically controlled, calibrated resistance trainer (Computrainer Lab^TM^ (Racermate, Seattle, WA, USA) was used throughout the study [16]; during the preliminary visit, to assess $\dot{V}O_{2\mathrm{peak}}$ as well as during the familiarization trial and experiments. The participants used their own road bicycle with a trainer-specific tire inflated to 110 psi. After the first 10 min of pedaling at an individualized workload, the resistance trainer was calibrated according to the manufacturer’s guidelines. The fixed-workload exercise period was completed in a cadence independent mode, i.e., the power output was maintained constant by the resistance trainer. Each participant was asked to select a combination of front/rear gears and use the same combination to complete both fixed-workload exercise periods. The TT was conducted in a cadence dependent mode, with the athletes self-selecting their gears. 

### 2.13. Fluid Balance

Net mass change from substrate oxidation during the 5 h fixed-workload exercise period was calculated as in Maughan et al. [17]. Carbohydrate and fat oxidation were computed from $\dot{V}O_{2}$ and respiratory exchange ratio and the equations of Péronnet and Massicotte [18]. For the TT, the $\dot{V}O_{2}$ and $\dot{\mathrm{VC}}O_{2}$ values were predicted for every kilometer based on the linear relationship between the workload and $\dot{V}O_{2}$ and $\dot{\mathrm{VC}}O_{2}$ values measured during the incremental test to exhaustion ($\dot{V}O_{2\mathrm{peak}}$ test). When the respiratory exchange ratio exceeded 1, it was considered that glycogen and glucose accounted for 80% and 20% of the substrate used, respectively [19]. Respiratory water losses were computed as in Mitchell et al. [20]. Sweat loss is the remaining fraction of the body mass lost after considering the water and gels consumption, urine production, metabolic mass loss, and respiratory water loss. Fluid balance during the experiments was computed with the following formula:

Fluid intake (kg) + metabolic water production (kg)—urine production (kg)—respiratory water loss (kg)—sweat loss (kg).

### 2.14. Local Sweat Sodium Concentration

Sweat was collected on the right forearm of the participants’ using 15 cm^2^ self-adhesive absorbent patches (Tegaderm + Pad, 3M, Maplewood, MN, USA) at 4 different times during the 5 h fixed-workload exercise period. Patches were applied at 20, 110, 200 and 270 min. They were removed from the skin with sterile tweezers before being saturated. Patches were centrifuged in 15 mL tubes to extract sweat, which was then frozen at −20 °C until analysis. Sweat sodium concentration was measured using the ion selective electrode technique (B-722 Laqua Twin, Horiba Scientific, Edison, NJ, USA). Whole-body sweat sodium concentration was estimated from local sweat sodium concentration according to Baker et al. [21]. The mean sweat sodium concentration during exercise was calculated as the average of the four samples.

### 2.15. Rectal and Skin Temperatures and Heart Rate

Rectal temperature was measured with a calibrated telemetric pill (CorTemp^TM^, HQ Inc, Palmetto, FL, USA) inserted just beyond the anal sphincter. Each pill was reused—after proper sterilization—up to 50 h [22,23]; a given pill was shared by several participants, but each participant exercised with the same pill. A total of 4 different pills were used during the study. Skin temperature was recorded on the left forearm, chest, thigh and calf with YSI 409 B probes (Yellow Springs Instrument, Yellow Springs, OH, USA) held in place with Hypafix adhesive tape (BSN medical, Hamburg, Germany). Mean skin temperature was calculated according to Ramanathan [24]. Heart rate was measured using a Garmin Premium chest electrode (Garmin, Olathe, KS, USA).

### 2.16. Perceptual Variables

Measurements were made of perceived heat stress (1–7 arbitrary scale) according to Dion et al. [2], exertion using the 6–20 Borg scale [25], thirst (1–11 arbitrary scale) and abdominal discomfort (1–5 arbitrary scale) according to Goulet et al. [26].

### 2.17. Capillary Blood Collection

Capillary blood samples were obtained with a finger prick (BD Microtainer, Mississauga, ON, Canada) as explained in Savoie et al. [27]. Blood (40 µL) was first collected with two HemoPoint H2 Microcuvettes (Alere, Waltham, MA, USA) for hemoglobin assessment (HemoPoint H2 photometer, Waltham, MA, USA) and then with a heparinized Minivette^TM^ (100 µL) (Sarstedt, Nümbrecht, Germany) after which the blood was transferred to a handheld blood analyzer (i-STAT, Abbott, Chicago, IL, USA) for hematocrit and sodium analyses. Plasma volume changes were calculated using the Dill and Costill equation [28].

### 2.18. Cramp Induction Protocol

The cramp induction protocol was performed inside the environmental chamber, with the participant sitting upright on a chair with his right thigh held firmly in place, the arms crossed over the chest and the right ankle in 30-degree of plantar flexion. A maximal voluntary isometric contraction of the plantar flexors was performed against a force plate (Vernier, Beaverton, OR, USA) for 3 s followed by a 2 min passive recovery period during which the subject remained sited with its leg and ankle held in place. In the instance of a muscle cramp, the experimenter stretched the participant’s plantar flexors by placing his ankle in a dorsal flexion. This took only a few seconds and was completed within the 2 min recovery period. The peak force production of 6 contractions was recorded during the preliminary visit; 3 were performed during the experiments. The presence or absence of a muscle cramp was assessed with the participant’s feedback and categorized as ‘no cramp’, ‘near cramp’ or ‘cramp’. A ‘near cramp’ was the sense that a cramp was going to occur but did not. 

### 2.19. Statistical Analyses

Data normality was assessed with Shapiro–Wilk tests. Paired sample t-tests were used to compare normally distributed variables assessed on only two occasions during the experiments, for instance the 20 km TT. The Wilcoxon rank sum test was used to compare total exercise time, time taken to urinate, pre-exercise urine specific gravity, pre-exercise urine osmolality and body water balance during the TT as these variables were not normally distributed. Two-way repeated measures analyses of variance (ANOVA) were performed to assess the effect of condition, time and their interaction on normally distributed variables that were measured on repeated occasions during experiments (blood sodium, $\dot{V}O_{2}$, substrates oxidation, sweat loss, plasma volume change, heart rate, rectal temperature, skin temperature, perceived exertion and perceived thirst). Linear mixed model analyses were performed for non-normally distributed variables (perceived heat stress and abdominal discomfort) and for a variable with missing data (peak force production of the plantar flexors). When required, post-hoc comparisons were performed using the false discovery rate procedure. Cramp categorization was analyzed with a chi-square test. Effect sizes were calculated for the cycling performance-related data using Cohen’s d (*d*) [29], with the following formula: *t*/√*n*. An effect size <0.20 was considered to be trivial and unsubstantial, between 0.21 and 0.49 small but substantial, between 0.50 and 0.79 moderate and >0.80 large and substantial. Based on a standard error of measurement (SEM) of 1.9% for the 20 km cycling TT performance [30], a power analysis (α = 0.05, β = 0.2) revealed that there was a 92% probability that the study would detect a treatment difference of 3.8% (twice the SEM value) between conditions. Statistical analyses were performed using the R software (R foundation for Statistical Computing, Vienna, Austria). A *p* value < 0.05 was accepted as the level of statistical significance. Data are presented as mean ± standard deviation (SD).

## 3. Results

### 3.1. Hydration State Prior to the Experiments and Environmental Conditions

Participants started both experiments in a well and similarly hydrated state, as evidenced by the lack of significant difference (all *p* > 0.05) between PFI and TDFI prior to starting the exercise periods for body mass, urine specific gravity, urine osmolality, hemoglobin level, hematocrit and pre-exercise heart rate (Table 1). Mean temperature (30.4 ± 0.2 vs. 30.3 ± 0.2 °C) and relative humidity (35.5 ± 0.7 vs. 35.8 ± 1.4%) of the environmental chamber during exercise with TDFI and PFI were similar (both *p* > 0.05).

### 3.2. Nutrition and Water Temperature

The same amount (*p* = 0.73) of energy gel was consumed between conditions, such that the intake of CHO and sodium, respectively, amounted to 341 ± 29 g and 2330 ± 196 mg for PFI, compared to 342 ± 33 g and 2333 ± 221 mg for TDFI. The rates of sodium intake with PFI and TDFI were, respectively, 418 ± 37 and 419 ± 40 mg·h^−1^. The temperature of the ingested water was 4.3 ± 0.3 °C at the beginning of every hour and 18.1 ± 1.3 °C at its end for the TDFI trial, for an average of 11.2 ± 0.7 °C.

### 3.3. Body Mass Loss and Fluid Balance

**Figure 2:**
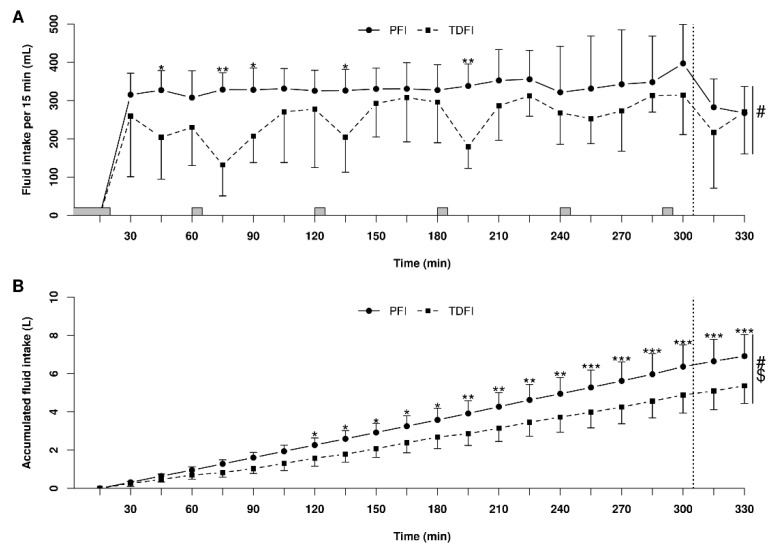
Patterns of fluid intake during the 5 h fixed-workload exercise period and the 20 km time-trial for the programmed (PFI) and thirst-driven fluid intake (TDFI) strategies. Fluid intake per 15 min interval (**A**) and accumulated fluid intake (**B**). * *p* < 0.05, ** *p* < 0.01, *** *p* < 0.001 for difference between conditions, # significant time effect (*p* < 0.001), $ significant interactions (*p* < 0.05). Vertical dotted line represents the onset of the time-trial. Shaded areas above the *x*-axis represent the moments when athletes did not have access to water. Values are means ± SD.

Fluid balance data for the 5 h fixed-workload exercise periods and the 20 km TTs are presented in Table 2. Relative body mass loss was not significantly different between TDFI and PFI both at the beginning (*p* = 0.22) and end (*p* = 0.10) of the TT. Body water balance was also not significantly different between conditions at the beginning (*p* = 0.19) and end (*p* = 0.09) of the TT. Fluid intake patterns utilized by the participants during PFI and TFI are shown in Figure 2A. Throughout exercise, more fluid was consumed every 15 min with PFI than TDFI, which led to more accumulated fluid intake over time with the former rather than the latter strategy (Figure 2B).

### 3.4. Sodium Balance

There was no difference in local sweat sodium concentration between the conditions (*p* = 0.83) with 66 ± 23 mmol·L^−1^ for PFI and 66 ± 24 mmol·L^−1^ for TDFI. Total whole-body sodium losses during the 5 h fixed-workload period were similar between conditions (*p* = 0.66) with 6315 ± 1357 mg (275 ± 59 mmol) and 6430 ± 1797 mg (280 ± 78 mmol) for PFI and TDFI, respectively. Considering the sodium consumed with the energy gels, the sodium deficits at the end of the 5 h fixed-workload exercise period were also similar (*p* = 0.67) with 4200 ± 1414 mg (183 ± mmol) for PFI and 4311 ± 1848 mg (188 ± 80 mmol) for TDFI.

### 3.5. Blood Sodium and Plasma Volume

**Figure 3:**
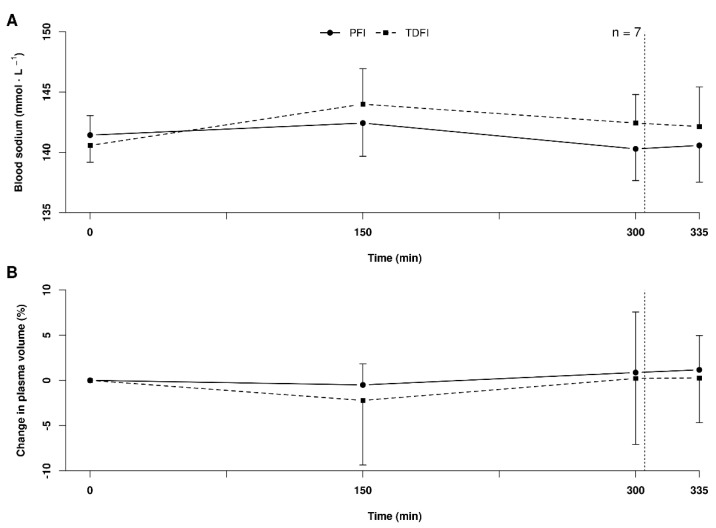
Blood sodium concentrations (**A**) and plasma volume variations (**B**) during the 5 h fixed-workload exercise period and at the end of the 20 km time-trial for the programmed (PFI) and thirst-driven fluid intake (TDFI) strategies. Vertical dotted line represents the onset of the time-trial. Values are means ± SD.

Due to a measurement error, the sodium values of one participant were excluded from the analysis. Figure 3A shows the changes in blood sodium concentration over time with PFI and TDFI. Blood sodium concentration was lower with PFI than TDFI during exercise, but the difference did not reach significance. The lowest blood sodium value measured with TDFI was 136 mmol L^−1^ (*n* = 1), compared to 137 mmol L^−1^ with PFI (*n* = 2) at the end of PFI. As demonstrated in Figure 3B, the plasma volume variations were similar between conditions (*p* = 0.67), with no time (*p* = 0.30) or interaction effects (*p* = 0.93).

### 3.6. Heart Rate, Rectal Temperature, and Skin Temperature

**Figure 4:**
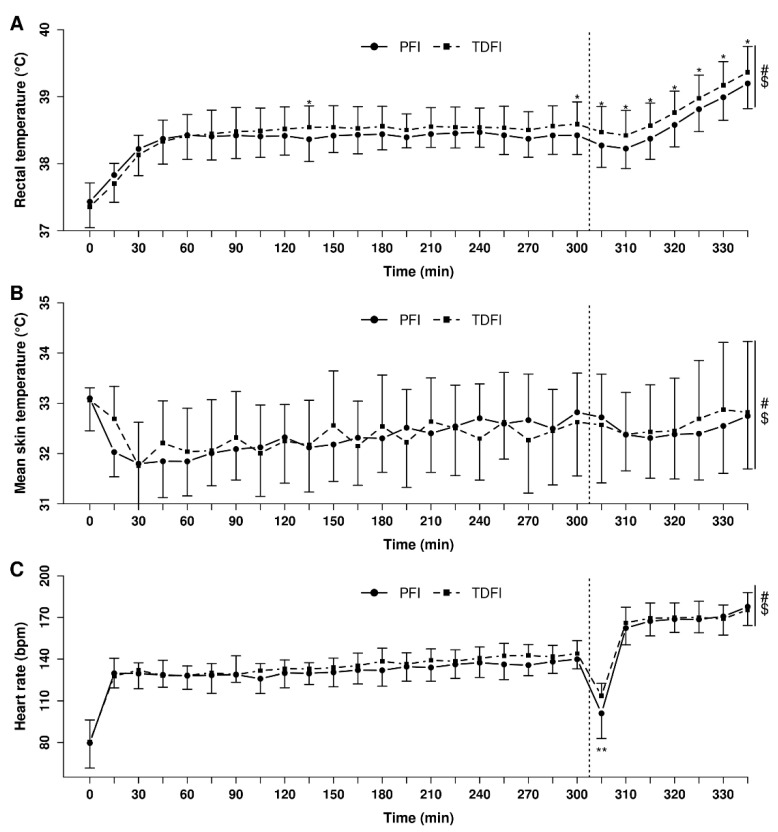
Heart rate (**A**), mean skin (**B**) and rectal temperatures (**C**) during the 5 h fixed-workload exercise period and 20 km time-trial for the programmed (PFI) and thirst-driven fluid intake (TDFI) strategies. * *p* < 0.05, ** *p* < 0.001 for difference between conditions, # significant time effect (*p* < 0.001), $ significant interaction (*p* < 0.05). Vertical dotted line represents the onset of the time-trial. Values are means ± SD.

Figure 4 depicts the changes in rectal temperature (A), mean skin temperature (B) and heart rate (C) over time during exercise with PFI and TDFI. An effect of time (*p* < 0.0001) and interaction (*p* < 0.0001), with a trend for a condition effect (*p* = 0.054), was observed for rectal temperature. Rectal temperature reached 38.4 ± 0.3 °C and 38.6 ± 0.3 °C at the end of the 5 h fixed-workload exercise period for the PFI and TDFI conditions, respectively (*p* < 0.05). At the end of the TT, rectal temperature was still lower (*p* < 0.05) with PFI (39.2 ± 0.4 °C) than TDFI (39.4 ± 0.4 °C). Mean skin temperature was similar between the conditions (*p* = 0.834), but an effect of time (*p* < 0.0001) and interaction (*p* < 0.05) was observed. There was no effect of condition on heart rate (*p* = 0.23), but an effect of time (*p* < 0.0001) and interaction was observed (*p* < 0.05).

### 3.7. Substrate Oxidation

Carbohydrate oxidation decreased over time (*p* < 0.05) from 2.0 ± 0.3 to 1.6 ± 0.4 g min^−1^, with no condition (*p* = 0.21) or interaction (*p* = 0.75) effects. Fat oxidation increased similarly over time (from 0.7 ± 0.2 to 0.9 ± 0.2 g min^−1^, *p* < 0.0001) between conditions (*p* = 0.43).

### 3.8. Perceptual Variables

**Figure 5:**
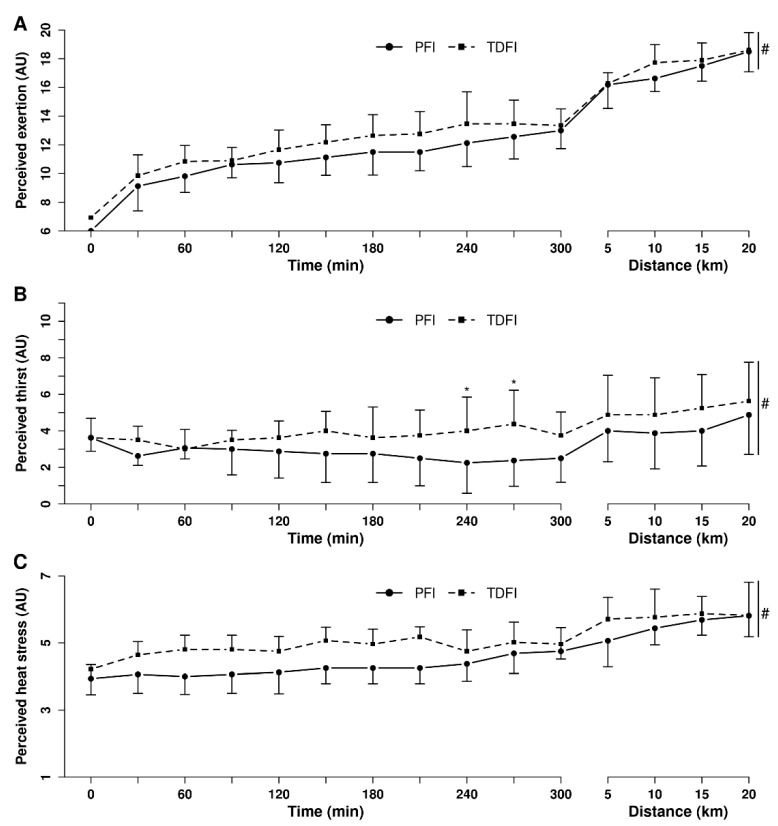
Perceived exertion (**A**), thirst (**B**) and heat stress (**C**) during the 5 h fixed-workload exercise period and the 20 km time-trial for the programmed (PFI) and thirst-driven fluid intake (TDFI) strategies. * *p* < 0.05 for difference between conditions, # significant time effect (*p* < 0.001). Vertical dotted line represents the onset of the time-trial. AU = arbitrary units. Values are means ± SD.

Figure 5 demonstrates the changes in perceived exertion (A), heat stress (B) and thirst (C) over time during exercise with PFI and TDFI. Perceived exertion and perceived heat stress increased similarly between conditions (both *p* > 0.05) over time (both *p* < 0.01), with no interaction effect (both *p* > 0.05). Perceived thirst increased over time (*p* < 0.0001), more with TDFI than PFI (*p* < 0.05), but no interaction was observed between conditions (*p* = 0.09). 

Abdominal discomfort did not change through time (*p* = 0.15), between conditions (*p* = 0.20) or differently through time between conditions (*p* = 0.86). The mean abdominal discomfort was 1.2 ± 0.5 and 1.0 ± 0.2 (1–5 scale) for PFI and TDFI, respectively; however, one participant suffered from moderate to considerable abdominal discomfort during the PFI trial and was unable to drink all the required water in the last 75 min of the 5 h fixed-workload exercise period. His perceived abdominal discomfort peaked at 3.5 (1–5 scale) after 240 min before getting back down to two for most of the TT during which this participant was able to drink the programmed amount of fluid, but still ended up with a body mass loss of 3.2% with PFI.

### 3.9. Exercise Intensity and Endurance Performance 

**Figure 6:**
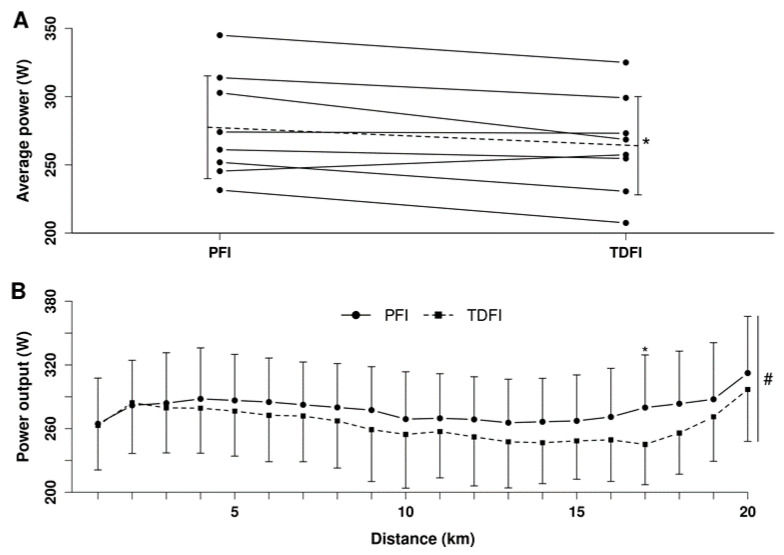
Average power outputs of each participant (**A**) and changes in power output (**B**) during the 20 km time-trial for the programmed (PFI) and thirst-driven fluid intake (TDFI) strategies. Dotted line represents the average of the 8 participants. * *p* < 0.05 for difference between conditions, # significant distance effect (*p* < 0.001). When required, values are reported as means ± SD.

Mean relative $\dot{V}O_{2}$ over the first 20 min of the fixed-workload exercise period was 59.5 ± 3.0% of $\dot{V}O_{2\mathrm{peak}}$ vs. 63.4 ± 4.1% at its end, with a time (*p* < 0.01), but no condition (*p* = 0.45) or interaction (*p* = 0.75) effects. Mean power output maintained during the fixed-workload exercise period was 183 ± 14 W. There was no difference (*p* = 0.89) in the mean TT power output between the first (271 ± 42 W) and second (270 ± 39 W) experimental trial, thereby indicating no order effect. As demonstrated in Figure 6A, mean power output for the 20 km TT was greater (*p* < 0.05) with the PFI (278 ± 41 W) than the TDFI (263 ± 39 W) (*d* = 0.93) condition. Accordingly, it took less time to complete the 20 km TT (*p* < 0.05) for PFI (31.8 ± 1.7 min) than TDFI (32.5 ± 1.9 min) (*d* = 0.89); however, when considering the difference in body mass, relative mean power output was similar (*p* = 0.15) between the PFI (3.9 ± 0.6 W·kg^−1^) and TDFI (3.8 ± 0.6 W·kg^−1^) condition. Only one participant performed better in the TDFI trial (Figure 6A). As shown in Figure 6B, mean power output production during the TTs varied according to conditions (*p* < 0.05) and distance (*p* < 0.0001), but no interaction was observed (*p* = 0.7). Total time taken to urinate was not different between conditions with 2.7 ± 3.1 min for PFI and 1.3 ± 1.5 min for TDFI (*p* = 0.11). Total performance time, including the breaks to urinate, was similar (*p* = 0.46) for both hydration strategies with 334.5 ± 3.1 min for PFI and 333.8 ± 2.0 min for TDFI.

### 3.10. Exercise-Induced Muscle Cramps

**Figure 7:**
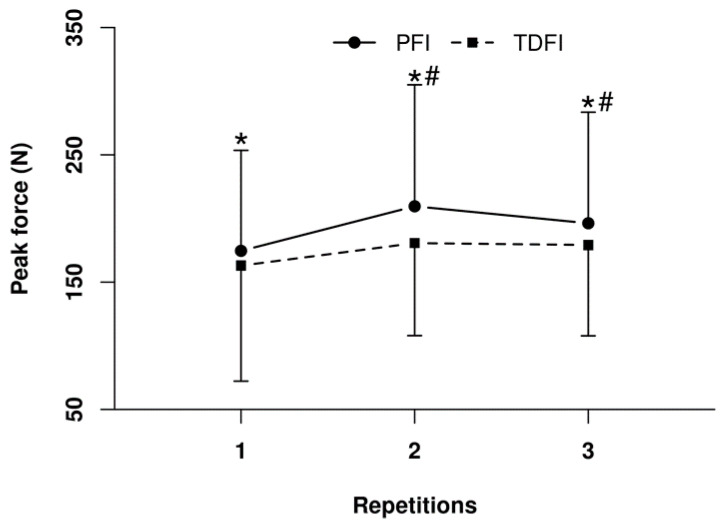
Peak force produced during the 3 s maximal voluntary isometric contraction of the right plantar flexors after the 20 km time-trial for the programmed (PFI) and thirst-driven fluid intake (TDFI) strategies. * *p* < 0.05 for difference between conditions, # *p* < 0.05 for difference with first repetition. Values are means ± SD.

Regarding the incidence of muscle cramps, 25% of contractions of the right plantar flexors led to a true cramp, but there was no difference (*p* = 0.75) between conditions for the prevalence. Nevertheless, as demonstrated in Figure 7, the mean peak force produced against the force plate was greater (*p* < 0.01) with PFI than TDFI for all three trials. Moreover, mean peak force production increased over time with PFI (*p* < 0.05), which was not observed with TDFI.

## 4. Discussion

The aim of the current study was to compare the effect of PFI and TDFI during prolonged cycling exercise. Contrary to one of our hypotheses, the results showed that PFI is the best strategy to optimize EP during a 20 km TT following 5 h of moderate intensity cycling in a warm environment. Indeed, PFI was associated with a large effect on EP with a 5% greater power output over TDFI; however, when the breaks to urinate were considered in the total exercise time, no significant difference in performance time was observed between hydration strategies. We had also hypothesized that the prevalence of EAMC immediately following exercise would be similar between drinking conditions. Our results showed that EAMCs were observed similarly with both hydration conditions. Finally, hyponatremia was not observed in either of the hydration conditions, as anticipated.

This was the first study to compare the effect of PFI and TDFI on cycling performance in a controlled environment during an exercise >2 h. There are some studies that have compared the effect of these hydration strategies on cycling endurance performance during shorter exercise duration in similar environmental conditions. Bardis et al. [31,32] observed no difference in EP between PFI and *ad libitum* drinking during a 30 km ride comprising three series alternating between 5 km fixed work rate periods at 50% of $\dot{V}O_{2\max}$ and 5 km TTs. Similarly, Dugas et al. [33] observed no change in endurance performance between PFI and *ad libitum* fluid consumption during an 80 km cycling TT. Finally, Perreault-Briere et al. [4] showed no difference between TDFI and PFI during a 60 min TT. Altogether, these results suggest that PFI incurs an EP advantage during prolonged exercise of 5 to 6 h in duration, but not during exercise <2 h. Work still needs to be performed to determine the effect of these hydration strategies on EP during exercises of 2–5 h in duration. 

The 5 h fixed-workload period was conducted at an average power output of 183 W; in comparison, international level professional road cyclists have been shown to maintain, on average, a power output of 192 W for 5 h during grand tours flat stages [34]. During the TT power output was significantly and substantially greater with PFI than TDFI. More work production with PFI should have led to a greater increase in rectal temperature during the TT, compared with TDFI, yet, the rate of increase in rectal temperature during the TT was similar between conditions. Moreover, the absolute difference in rectal temperature (0.2 °C) between drinking conditions was similar at the onset and end of the TT. A greater rate of heat loss with PFI is unlikely as the sweat rate was similar between conditions as well as the impact of convection. We propose that the favorable effect of PFI on rectal temperature may have been due to a greater heat sink effect from cool fluid intake. Heart rate and the relative change in plasma volume were similar between conditions during the TT. This implies that cardiac output was also alike between the conditions during the TT. Because of the lower heat strain with PFI a greater portion of the cardiac output may have been devoted to the working muscles, thereby potentially explaining the difference in TT performance.

Hyperthermia per se may contribute to central fatigue during prolonged exercise and affect motor activation directly in the brain [35]. It is important to point out that the observed difference in rectal temperature was relatively small and that it did not reach the critical value of 40 °C for any athlete [36,37]. Combined together, these observations might suggest that hyperthermia-associated decrease in motor activation is unlikely to have played a causal role for the lower performance with TDFI. To this effect, Périard et al. [38] did not observe a difference in force production or on voluntary activation despite a difference in rectal temperature of 0.8 °C (39.8 °C vs. 39.0 °C) following a 40 km cycling TT in warm (35 °C) or temperate (20 °C) ambient conditions. However, we observed that peak force production of the plantar flexors during the maximal voluntary isometric contractions following the TTs was significantly lower with TDFI than PFI, which indicates that an effect of the core temperature on the voluntary activation of the active muscles cannot be ruled out. It is possible that when sustained over several hours even a small difference in core temperature may reduce the efficacy of the central nervous system to recruit muscle fibers potentially to prevent biological harm [39].

As expected, EAMC did occur with both of the drinking conditions, but their prevalence was not significantly different between drinking conditions. In association, we observed no significant difference in blood sodium concentration, changes in plasma volume or on body water balance between trials and neither the PFI nor TDFI led to a serious dilution of blood sodium concentration or to severe dehydration. Therefore, considering that EAMC were still observed with each drinking condition, our results lend more support to the altered neuromuscular control hypothesis for the aetiology of EAMC [40,41], for if substantial dehydration or electrolyte losses/dilution are key players then no EAMC should have occurred in the current exercise scenario.

The rates of water intake during exercise were 0.27 and 0.21 mL·kg^−1^·min^−1^ with PFI and TDFI, respectively. These values are within the 0.14–0.27 mL·kg^−1^·min^−1^ recommended range for cycling exercise lasting over 2 h [42]. For reference, professional road cyclists drank 0.30 mL·kg^−1^·min^−1^ during one stage of a 6-day stage race when the air temperature was 33 °C [43]. It must be noted that reductions in fluid intake were observed (Figure 2A) for the first 15 min of the second, third and fourth hour with TDFI. This may have been due to the greater thirst-quenching effect of colder water [44] that was provided at the beginning of each hour. More importantly though, participants were also unable to drink during the first 5 min of every hour due to the measurement of oxygen consumption, and it appears that they did not naturally compensate for this interruption with TDFI. No athletes developed hyponatremia despite the high rates of fluid intake sustained for several hours. Moreover, whole-blood sodium levels were not significantly different between PFI and TDFI, but this likely came at the expense of more cycling breaks that negated the time gains provided with PFI during the TT. Indeed, we posit that the additional urine produced with PFI was likely required to avoid further dilution of sodium and avoid hyponatremia. Apparently, the exercise intensity and thermal stress during the fixed-workload period were low enough to allow for proper kidney function by maintaining sufficient renal blood flow and glomerular filtration rate [45]. Hourly whole-body sweat sodium losses during the 5 h fixed-workload exercise were comparable with published normative data for endurance athletes [46]. Hence, a relatively small amount of sodium intake during prolonged exercise is sufficient to maintain whole-blood sodium concentration within a normal range [47] in athletes with average losses of sodium through sweat. Overall, our findings indicate that the rates of fluid consumption with PFI and TDFI during prolonged moderate intensity cycling exercise in the heat are reasonable, within the recommended ranges and unlikely to cause hyponatremia in trained athletes consuming sodium at a rate of 420 mg·h^−1^.

EP was improved with PFI only when examining the absolute power output maintained during the TT; however, there were no differences between hydration strategies when considering the total performance time including the breaks to urinate and for the power output relative to body mass. Finally, physiological and perceptual differences were relatively trivial between conditions during the fixed-workload cycling exercise. These observations suggest that the ideal hydration strategy to be used by athletes may be situation-dependent. (1) Programmed fluid intake may be more advantageous for performance, whereas both hydration strategies may confer the same advantage during extensive training. (2) Having to stop to urinate may have serious consequences in terms of performance during competitions, whereas during training it is inconsequential. (3) Given the lack of difference in relative power output between drinking conditions, PFI may not be as advantageous as TDFI for a competition featuring an uphill finish or in activities where body mass carriage is important. Since the implementation of TDFI is easier than PFI, assessing the ability of an athlete to maintain proper fluid balance and EP with the former strategy may be a great starting point to determine the ideal hydration strategy for this athlete.

This study has some limitations. It may have been underpowered to detect significant differences between conditions for variables that were repeatedly measured over time and for body water balance for which some of the variables included in its computation varied substantially between participants. Trials were scheduled to minimize variations in fitness level or heat acclimation, but such variations cannot be completely ruled out as the delay between experimental trials was long enough to allow such adaptations to take place. The nature of the intervention did not allow for a blinding of the participants. Therefore, a placebo or nocebo effect for one condition or the other could partly explain the difference in performance between hydration strategies. Nonetheless, the large effect size observed for the change in performance combined with the significant difference in rectal temperature despite a lack of variation in perceived exertion and heart rate between conditions during the TT do not point in that direction. Sodium intake can have an impact on thirst-driven fluid consumption during exercise [48]. Therefore, different patterns of fluid intake and, hence, potential effect on EP, may have been observed had we provided higher or lower amounts of sodium [49]. As discussed, the effectiveness of PFI may be related to the temperature of the ingested fluid through a heat sink effect. Different results may have been obtained had we provided warmer water. Furthermore, athletes had unlimited access to water during the TDFI condition, which may explain why they replaced a substantial part of their sweat losses. Our findings may have been different if participants had limited access to fluid during exercise, as is commonly the case during real-world exercise conditions. Finally, it is important to consider the environmental conditions, the population, and the exercise intensity when interpreting these results, which may not apply to environments cooler than 30 °C or with relative humidity far greater than 35%, to athletes of inferior level or to prolonged even-paced efforts.

## 5. Conclusions

PFI is a better hydration strategy than TDFI to optimize performance during a 20 km cycling TT following 5 h of moderate intensity cycling in a warm environment. The results of this study confirm that the current guidelines regarding hydration—recommending PFI to prevent a body mass loss >2% [5]—are appropriate to maximize EP at the end of a 5 h cycling exercise; however, urine production and potential breaks in exercise is of concern when using this hydration strategy. Therefore, endurance athletes cycling in the heat for 5 h are advised to plan their fluid intake to limit their body mass loss <2% in scenarios where urine production is not a concern. Despite this, during prolonged training, both hydration strategies appear equally effective in maintaining proper physiological responses and perceptual sensations.

## Figures and Tables

**Table 1:** Pre-exercise body mass, urine specific gravity, urine osmolality, hemoglobin level, hematocrit and heart rate for the programmed (PFI) and thirst-driven fluid intake (TDFI) strategies. Data are means ± SD.

Variables	PFI	TDFI	*p*
Body mass (kg)	71.6 ± 5.8	70.8 ± 5.5	0.16
Urine specific gravity (g·mL^−1^)	1.014 ± 0.011	1.012 ± 0.009	0.68
Urine osmolality (mOsm·kg^−1^)	615 ± 398	522 ± 305	0.47
Hemoglobin (g·dL^−1^)	16.0 ± 0.5	16.2 ± 0.7	0.17
Hematocrit (%)	44.9 ± 1.4	45.0 ± 4.1	0.92
Heart rate (bpm)	80 ± 18	80 ± 16	0.67

**Table 2:** Fluid balance data during the 5 h fixed-workload exercise period and the 20 km time-trial for the programmed (PFI) and thirst-driven fluid intake (TDFI) strategies.

	5 h Fixed-Workload		20 km Time-Trial		Total	
Variables	PFI	TDFI	PFI	TDFI	PFI	TDFI
Body mass loss (kg)	0.7 ± 0.9	1.3 ± 0.7	0.4 ± 0.3	0.5 ± 0.3	1.2 ± 0.8	1.8 ± 0.6
Body mass loss (%)	0.9 ± 1.2	1.9 ± 0.9	0.6 ± 0.4	0.6 ± 0.3	1.5 ± 1.0	2.5 ± 0.9
Water intake (mL)	6366 ± 1131	4880 ± 941 *	550 ± 122	487 ± 204	6916 ± 1124	5367 ± 925 *
Water intake (mL·kg body mass^−1^)	90 ± 19	70 ± 13 *	8 ± 2	7 ± 3	98 ± 20	77 ± 13 *
Urine production (mL)	1490 ± 937	638 ± 806 *	62 ± 84	52 ± 67	1552 ± 979	690 ± 788 *
Urine production (mL·kg body mass^−1^)	21 ± 14	9 ± 11 *	0.9 ± 1.3	0.8 ± 0.9	22 ± 14	14 ± 11 *
Metabolic mass loss (g)	204 ± 26	184 ± 50	50 ± 12	57 ± 7	254 ± 32	242 ± 55
Metabolic water production (g)	598 ± 35	581 ± 44	86 ± 8	94 ±12	684 ± 39	675 ± 44
Respiratory water loss (mL)	580 ± 43	570 ± 48	77 ± 4	81 ± 10	657 ± 45	651 ± 48
Body water balance (mL)	−358 ± 900	−990 ± 807	−344 ± 251	−402 ± 316	−703 ± 817	−1392 ± 692
Sweat loss (mL)	5253 ± 549	5243 ± 712	841 ± 127	850 ± 199	6094 ± 607	6093 ± 611
Sweat rate (mL·h^−1^)	1051 ± 110	1049 ±142	1587 ± 227	1581 ± 443	1102 ± 107	1099 ± 107

* *p* < 0.05 for difference with PFI. Data are means ± SD.

## Data Availability

The data will be made available from the corresponding author upon reasonable request.
